# Enhancing protein signal detection in asexual and viviparous pea aphids: A guided protocol for tissue dissection and proteinase K treatment

**DOI:** 10.1016/j.mex.2024.102982

**Published:** 2024-09-27

**Authors:** Chun-wei Lai, Gee-Way Lin, Wen-Chih Lee, Chun-che Chang

**Affiliations:** a Laboratory for Genomics and Development, Department of Entomology, College of Bio-Resources and Agriculture, National Taiwan University (NTU), Taipei, Taiwan; b Genome and Systems Biology Degree Program, NTU, Taipei, Taiwan; c College of Medicine, Taipei Medical University, Taipei, Taiwan; d Institute of Biomedical Sciences, Academia Sinica, Taipei, Taiwan; e Institute of Biotechnology, College of Bio-Resources and Agriculture, NTU, Taipei, Taiwan; f Research Center for Developmental Biology and Regenerative Medicine, NTU, Taipei, Taiwan; g International Graduate Program of Molecular Science and Technology, NTU, Taipei, Taiwan; h Master Program for Plant Medicine, NTU, Taipei, Taiwan; i Taiwan Aphid Genomics Consortium, MK Innovation Hall, NTU, Taipei, Taiwan

**Keywords:** Antibody staining, Tissue permeability, Insect ovarioles, Salivary glands, Parthenogenetic viviparity, *Acyrthosiphon pisum*, Aphid-specific tissue dissection and whole-mount immunostaining

## Abstract

Aphids, as hemipteran insects, reproduce via parthenogenesis and viviparity, resulting in rapid and exponential offspring production. To investigate the molecular mechanisms underlying parthenogenetic viviparity in asexual aphids, precise protein detection through immunostaining is essential. Our previous research demonstrated the need for proteinase K (PK) treatment to improve tissue permeability, enabling antibodies targeting the germ-cell marker Ap-Vas1 to access gastrulating and later-stage embryos. However, optimal PK digestion protocols have not been thoroughly explored. In this study, we propose strategies to optimize PK digestion conditions for early, middle, and late-stage pea aphid embryos, which have varying tissue thicknesses. Additionally, we extend the application of PK treatment to salivary glands, a representative somatic tissue, by optimizing conditions for antibody penetration against the salivary gland marker C002. To enhance spatial precision in signal detection, we provide a detailed protocol for tissue dissection specific to pea aphids, focusing on the preservation of tissue integrity. These comprehensive guidelines, covering tissue dissection and PK titration, are expected to improve the specificity and intensity of protein signals in pea aphids and other aphid species.

• Provide aphid-specific dissection methods to obtain intact embryos and salivary glands.
• Present strategies for optimizing PK treatment conditions across different tissue types.

## Graphical abstract

**Figure:**
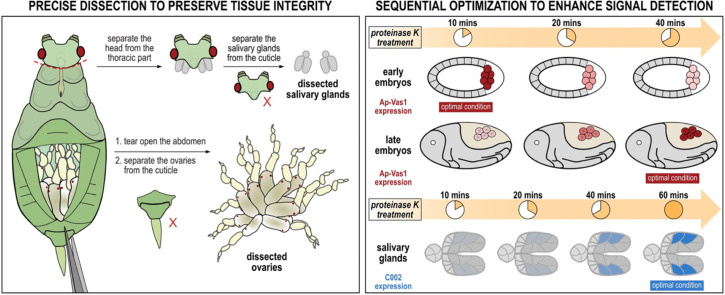

Specifications table

**Table:** 

Subject area:	*Biochemistry, Genetics and Molecular Biology*
More specific subject area:	*Developmental Biology*
Name of your method:	*Aphid-specific tissue dissection and whole-mount immunostaining*
Name and reference of original method:	*G.W. Lin, C-c. Chang, Identification of critical conditions for immunostaining in the pea aphid embryos: increasing tissue permeability and decreasing background staining, J. Vis. Exp.* (*2016*) *e53883,*doi:10.3791/53883M.
Resource availability:	*See Table 1*

## Background

Aphids, a family of millimeter-sized hemipteran insects, are notorious agricultural pests. Their sap-extracting feeding behavior causes plant discoloration and leaf curling [1,2]. Moreover, aphids act as vectors for plant viruses, transmitting these pathogens from infected plants, which can result in crop illnesses or even death [3]. Their rapid asexual (parthenogenetic) reproduction and viviparity further amplify their agricultural impact [4].

Unlike sexual and oviparous insects, where embryos develop from fertilized eggs laid by females, asexual and viviparous aphids develop embryos within multiple ovarian tubules (ovarioles) that collectively form an ovary [4,5]. This unique reproductive phenomenon permits the study of aligned developmental processes [6]. Methods for detecting mRNA and protein are essential for understanding developmental mechanisms at the molecular level, contributing to aphids becoming a rising model organism [7]. Previously, we established a robust platform for whole-mount *in situ* hybridization (WISH) to detect mRNA expression in the asexual pea aphid *Acyrthosiphon pisum* [8,9], the first aphid species to have its genome sequenced [10]. When performing WISH on pea aphid embryos, we found that omitting the proteinase K (PK) treatment step did not significantly affect signal intensity [8]. This suggests that the antisense riboprobes can penetrate aphid tissues without the need to enhance permeability through PK digestion.

In contrast to WISH, detecting protein expression in the aphid embryos did require PK treatment [11]. In pea aphid embryos, starting from the gastrulation stage, antibody penetration—including targeting the germline marker Ap-Vas1 (formerly ApVas1) [12]—was significantly hindered without PK digestion. Treating embryos with PK (1 µg/mL for 10 min) allowed for specific detection of Ap-Vas1 in germ cells [11]. Nevertheless, in mature embryos with cuticles, signal detection was minimal. To enhance tissue permeability in these embryos, we increased the concentration and duration of PK digestion. During optimization, we discovered that doubling the PK concentration or using even higher amounts did not improve antibody penetration into embryos with cuticles. Moreover, the structure of early-stage embryos was compromised by high-concentration of PK within as little as five minutes. Meanwhile, staining signals were barely detectable, suggesting that the target antigens had been digested by PK.

When extending PK incubation time beyond 10 min at the original concentration (1 µg/mL), we observed a significantly improvement in Ap-Vas1 signal intensity in embryos before cuticle formation. Additionally, we found that early, middle, and late embryos each have their own optimal PK treatment conditions. Therefore, developing a strategy-based protocol for optimizing PK treatment is essential for effective immunostaining, in contrast to most protocols that only provide the final optimized conditions [13,14].

Maintaining tissue integrity while ensuring permeability is crucial for accurate detection of protein expression [15,16]. Therefore, we have provided detailed instructions tailored for asexual pea aphids on dissecting ovarioles and salivary glands, which represent germline and somatic tissues, respectively. Since tissue dissection precedes antibody staining, our protocol outlines the dissection steps followed by PK treatment. Although these procedures were performed on the pea aphid *A. pisum*, they may also be applicable to other aphid species.

## Method details

### Materials

**Table 1:** List of materials along with their sources and identifiers.

Material	Source	Identifier
4′,6-Diamidino-2-phenylindole (DAPI)	Thermo Scientific	Product No 62248
*Acyrthosiphon pisum*	NTU strain [17]	Not Available (N/A)
Antiserum against Ap-Vas1	Chang Lab, NTU [12]	N/A
Antiserum against C002	Chang Lab, NTU	N/A
Biotinylated Goat Anti-Rabbit IgG antibody	Vector Laboratories	BA-1000–1.5
DIG Wash and Block Buffer Set	Sigma-Aldrich	Cat. No 11585762001
Paraformaldehyde	Sigma-Aldrich	Product No 158127
Phosphate buffered saline (PBS)	Bioman (10X PBS, pH 7.4)	Cat. No PBS105000
Proteinase K	BioShop	PRK403
SIGMAFAST 3,3′diaminobenzidine (DAB) with metal enhancer (tablets)	Sigma-Aldrich	Product No D0426
Triton X-100	Sigma-Aldrich	Product No X100
VECTASTAIN Elite ABC—HRP Kit, Peroxidase (Rabbit IgG)	Vector Laboratories	PK-6101

### Recipes of solutions

**Table 2:** Recipe for 4 % paraformaldehyde.

Composition	Final concentration	Amount
20 % Paraformaldehyde (w/v)	4 %	1 mL
Sterilized 1X PBS	N/A	4 mL
Total	4 %	5 mL

**Caution:** The paraformaldehyde is classified as a carcinogen. Always wear gloves to prevent skin contact.

***Note:*** Prepare freshly each time before use.

**Table 3:** Recipe for PBSTx.

Composition	Final concentration	Amount
Sterilized 1X PBS	N/A	50 mL
Triton X-100	0.2 %	100 µL
Total	0.2 %	50 mL

**Table 4:** Recipe for blocking reagent.

Composition	Final concentration	Amount
10X Blocking reagent	1X	5 mL
1X Maleic acid	0.9X	45 mL
Total	1X	50 mL

***Note:*** Reagents are components of the DIG Wash and Block Buffer Set from Sigma-Aldrich. Prepare freshly each time before use.

### Experimental procedures

#### Cultivation and breeding of asexual pea aphids

The asexual pea aphids, designated as the NTU strain (Table 1) [17], were supplied by Dr. Mei-Hua Kuo from National Chung Hsing University in 2004 and have been consistently cultivated in our laboratory ever since. The NTU strain feeds on the garden peas *Pisum sativum*, propagating parthenogenetically and viviparously in a growth chamber under a long-day photoperiod (16-hour light/8-hour dark) at 20 °C. The sexual phenotype could not be induced under a short-day photoperiod (12-hour light/12-hour dark) at 15 °C [18], indicating that the NTU strain might be obligately parthenogenetic [19]. The robust protocol for laboratory maintenance of the pea aphids, which has not been reported previously, is described below:

1. Soak pea beans in water until the small sprouts, known as hypocotyls, become visible. (Fig. 1A and A’). Change water daily to prevent bacterial growth.

**Fig. 1:**
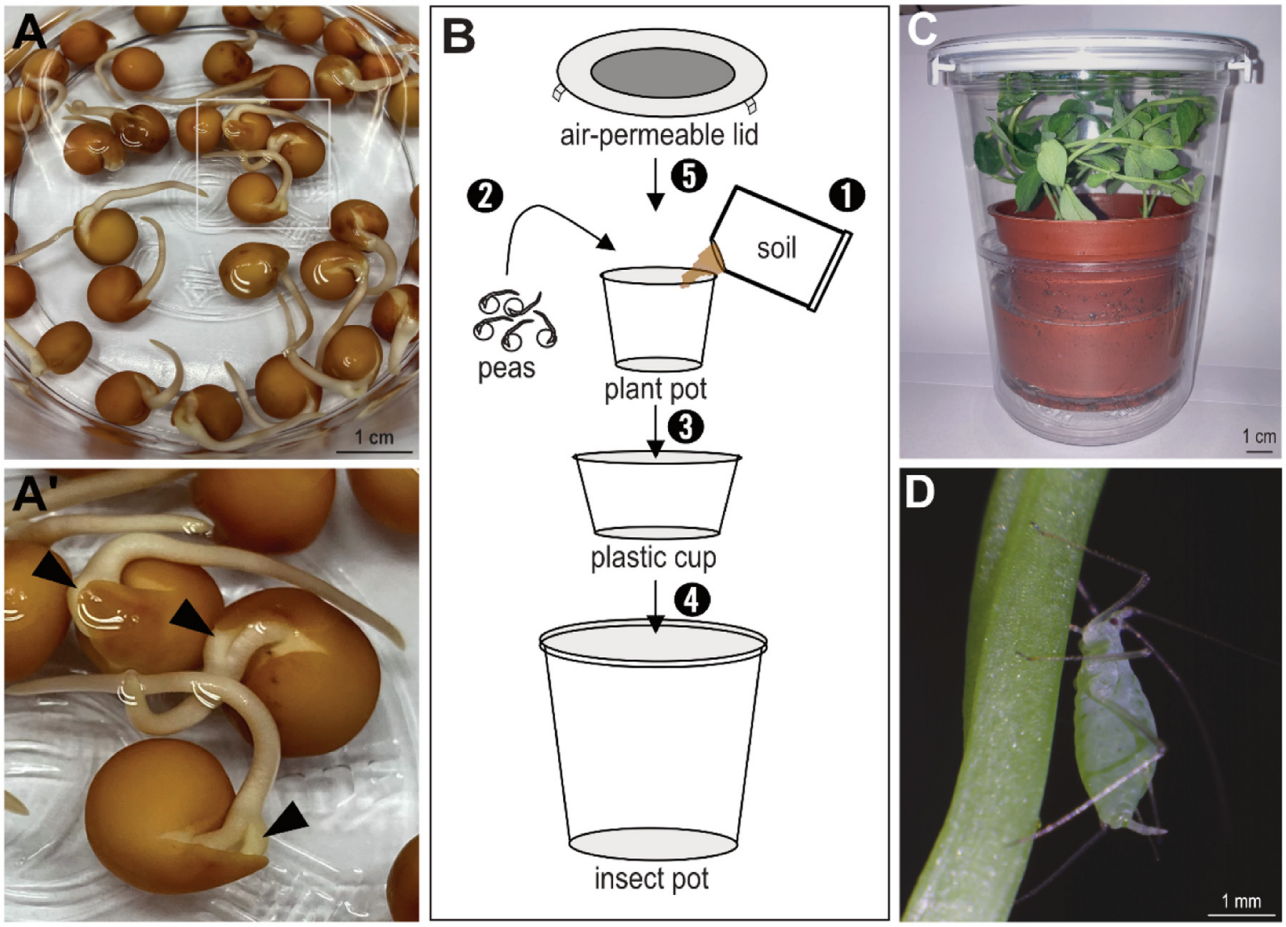
Setup of rearing apparatus for cultivating asexual pea aphids. (A) Pea beans undergo hydration in distilled water to initiate germination. (A') Magnified view of three pea beans illustrates the onset of germination with visible hypocotyls (black arrowheads), indicating readiness for sowing. (B) The assembly of the apparatus is depicted in multiple steps: step 1, filling a plant pot with soil; step 2, placing germinated peas in the soil; step 3, placing the plant pot into a plastic cup; step 4, inserting both into an insect pot; step 5, sealing the top of the insect pot with an air-permeable lid. (C) Water the growing peas by filling half of the plastic cup with distilled water. As the peas grow and reach the lid, they become suitable hosts for pea aphids. (D) An adult pea aphid is captured in the act of feeding on the pea plant using its stylet mouthpart.
  **Note:** After a 5-day incubation at room temperature, the emergence of exposed hypocotyls can be readily observed.
2. Prepare a plant plot with 9 cm in diameter and 7 cm in height. Fill the plant pot with soil up to two-thirds of its height. Next, plant 5 sprouting seeds beneath the soil surface (Fig. 1B, steps 1–2).
3. Prepare a plastic cup with 9.5 cm in diameter and 5.5 cm in height. Place the soil-filled pot inside the plastic cup (Fig. 1B, step 3). Water can be filled in the plastic cup to keep the soil moist.
4. Prepare an insect pot with 12.5 cm in diameter and 15 cm in height. Place the plastic cup inside the insect pot and secure an air-permeable lid to complete the aphid-rearing apparatus (Fig. 1B, steps 4–5).
  **Note:** An air-permeable lid can prevent aphids from escaping while allowing air to circulate.
5. Once the pea plants grow to reach the lid, transfer pea aphids onto the pea plants (Fig. 1C).
6. Use a paintbrush to transfer four adult aphids onto the pea plants, allowing them to commence feeding on plants and establish a colony (Fig. 1D). After transferring the aphids, place the apparatus in a growth chamber and set a long-day photoperiod at 20 °C as the growing condition.
7. To ensure a continuous supply of host plants, start another round of pea plant cultivation.

#### Dissection of ovaries from the asexual pea aphids

**Fig. 2:**
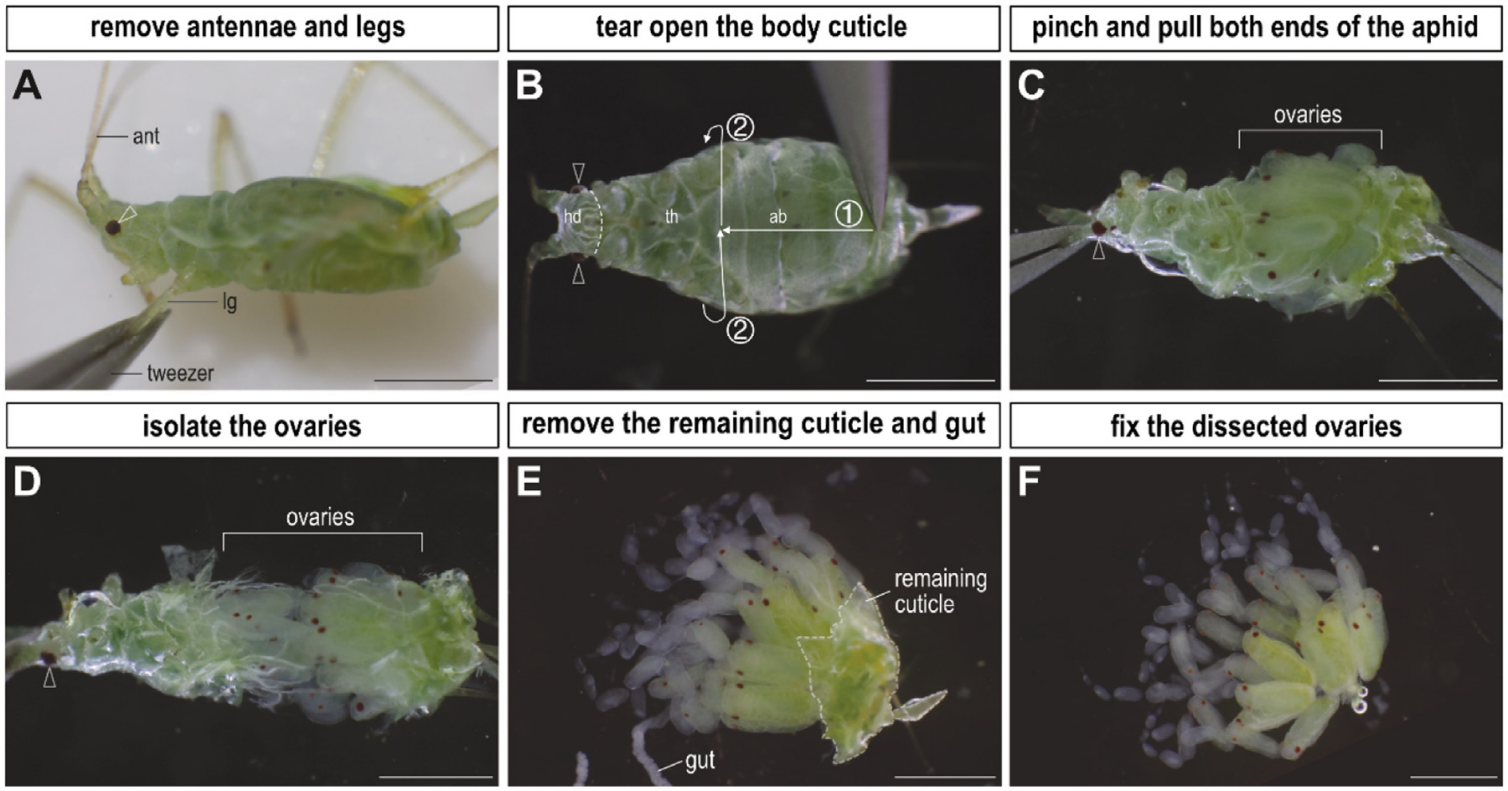
Dissection of ovaries from an asexual pea aphid. The pea aphid is positioned with its head to the left, and its eyes are marked by white hollow arrowheads. The entire dissection is conducted using a pair of fine tweezers. (A) Begin by removing the legs and antennae on a tissue paper. This panel illustrates the process of leg removal. (B) Submerge the aphid in a 4 % paraformaldehyde solution with the ventral side facing upward. Next, tear open the abdominal cuticle following the guidance of arrow directions (steps 1 and 2). (C) Pinch both ends of the aphid and gently pull them in opposite directions. (D) Continue pulling both ends until the ovaries are fully isolated. (E) Eliminate the remaining cuticle (encircled by a white dotted line) and the gut tissues adhering to the ovaries. (F) After removing all tissues attached to the ovaries, the dissection process is concluded. The ovaries are now prepared for fixation. Abbreviations: ab, abdomen; ant, antenna; hd, head; lg, leg; th, thorax. All images have scale bars of 1 mm.

In asexual pea aphids, similar to other insects, a pair of ovaries is located within the abdomen. Due to the distinctive characteristic of asexual viviparity, developing embryos are housed within egg chambers along ovarioles [1,4]. Fig. 2 provides a visual guide to the dissection approach and the structure of ovarioles. Details are further described as follows:

1. To initiate the aphid dissection process, place the aphid on a piece of tissue paper and use tweezers to remove the antennae and legs (Fig. 2A). This step is essential to prevent these appendages from damaging the delicate ovaries.
2. Submerge the aphid in a dissection dish filled with a 4 % paraformaldehyde (PFA) solution (Table 2). Once the aphid is fully immersed beneath the surface, expose its ventral side, tear open the cuticle from posterior abdomen to thorax (Fig. 2B, step 1), and peel the cuticle circularly (Fig. 2B, step 2).
3. With tweezers, grasp both ends of the aphid and apply a steady pulling force (Fig. 2C). Continue pulling until the ovaries have been extracted from the body cavity (Fig. 2D).
4. Remove the remaining cuticles and guts attached to the ovaries (Fig. 2E). Following this, the dissection of ovaries is complete and ready for further fixation (Fig. 2F).

#### Dissection of salivary glands from the asexual pea aphid

**Fig. 3:**
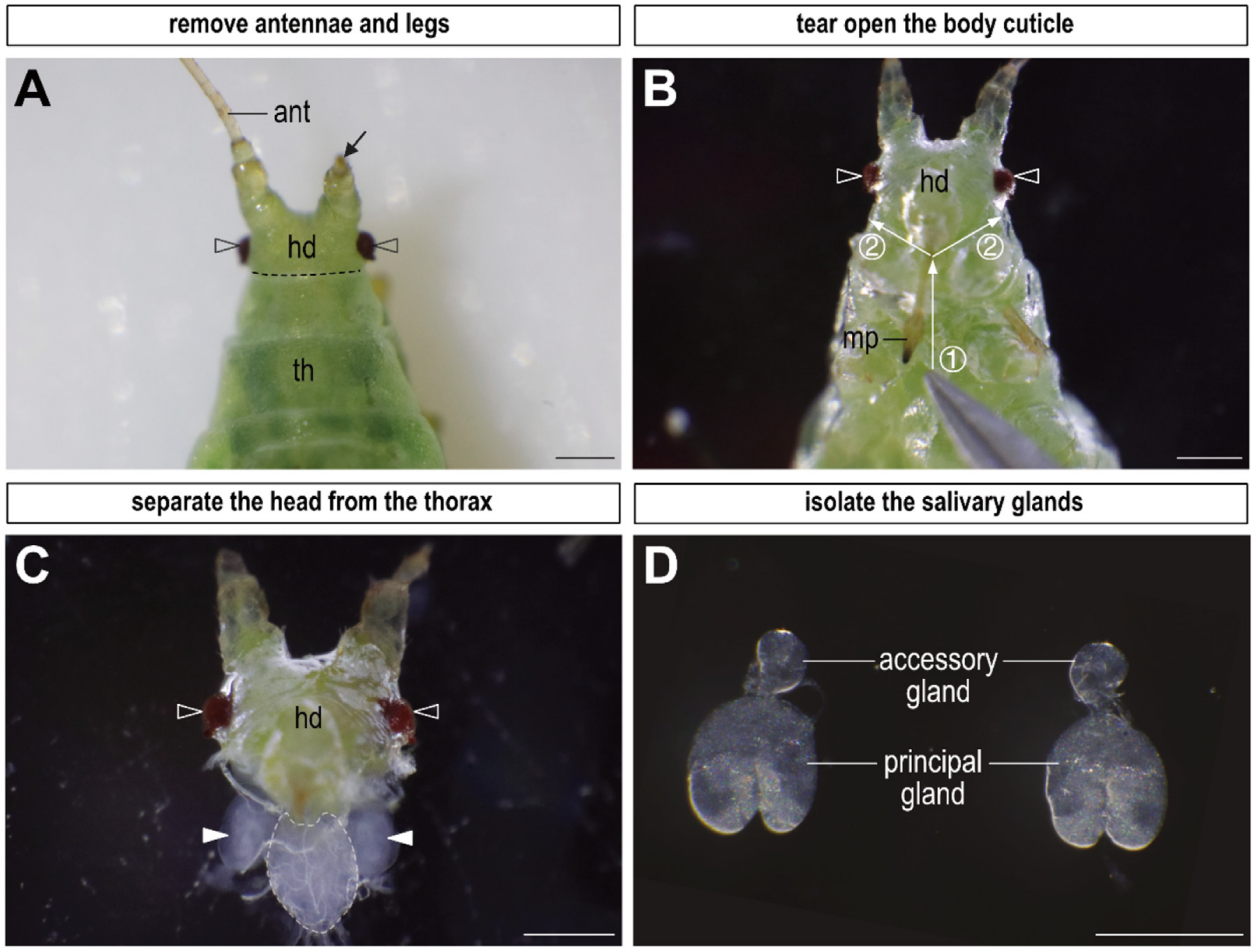
Dissection of salivary glands from an asexual pea aphid. The pea aphid is positioned with its head upwards, and its eyes are marked by hollow arrowheads. The entire dissection process is conducted using a pair of fine tweezers. (A) Begin by removing the legs and antennae on a tissue paper. This panel illustrates the process of antennae removal. The arrow indicates the base of the removed antenna. (B) Place and hold the aphid with the ventral side upward in a 4 % paraformaldehyde solution. Subsequently, tear open the thoracic cuticle following the indication of white arrows in order (steps 1 and 2). (C) Separate the head from the thorax, and the salivary glands (arrowheads) are found attached to the ganglion (encircled by a white dotted line). (D) Isolate a pair of salivary glands from the head. One of the paired salivary glands is composed of two units: the principal gland and the accessory gland. Abbreviations: ant, antenna; hd, head; mp, mouthpart; th, thorax. All images have scale bars of 0.25 mm.

In addition to the ovaries, we focus on detecting protein expression in other tissues in the pea aphid, including the salivary glands, a singular pair of tissues located between the head and thorax [20]. Fig. 3 provides a visual guide to the dissection technique and the structural of salivary glands. Details are further described as follows:

1. Remove the antennae and legs (Fig. 3A), as mentioned in the ovary dissection section.
2. Place the aphid in a dissection dish and submerge it in a 4 % PFA solution. Once fully immersed, carefully tear open the thoracic cuticle starting from the second thoracic segment towards the head (Fig. 3B, step 1), and proceed to open the cuticle around the margin between the head and thorax (Fig. 3B, step 2).
3. Separate the head from the thorax and carefully clean the sclerites surrounding the base of the head to reveal a pair of salivary glands attached to the ganglion (Fig. 3C).
  **Tip:** While the salivary glands are not isolated at this step, it is acceptable for the complex of head, salivary glands and ganglion to undergo the following process. The salivary glands can be eventually isolated after the completion of immunostaining.
4. Isolate the salivary glands from the ganglion. Within a detached salivary gland, two distinct components can be observed: a principal gland and an accessory gland. Following this step, the dissection of salivary glands is complete and ready for further fixation (Fig. 3D).
  **T**i**p:** The isolation of salivary glands from the ganglion is an optional step. If the isolated salivary glands are utilized, exercise with extra care during subsequent buffer changes, as these small glands are susceptible to loss during the process.

#### Pre-treatment of samples

Optimal conditions for sample pre-treatments, such as fixation, permeabilization, and endogenous background reduction, are crucial for the successful detection of protein expression in animal tissues. Fixation plays an important role in maintaining the integrity of cell morphology and tissue structure. Meanwhile, fixatives like paraformaldehyde can suppress the activity of endogenous proteinases, preventing the degradation of the sample [21]. Permeabilization is performed to facilitate antibody penetration into the fixed cells or tissues. This process is commonly achieved by adding suitable detergents into buffer solutions, such as Triton X-100 in PBS (PBSTx), where a detergent would disturb the lipid-based membrane integrity and make pores for antibodies to enter [22]. Before signal detection, reducing endogenous background is also a necessary step to increase signal-to-noise ratio for a better staining result. For example, endogenous peroxidase activity can be inactivated by methanol treatment, preventing the interference of background signals from endogenous peroxidase to the specific ones from the peroxidase conjugated to the secondary antibody [23].

Nevertheless, for some tissues, particularly those with increased thickness, treatment with proteinase K (PK) is additionally required to enhance tissue permeability, allowing antibodies to penetrate into target cells [14,24]. The guiding steps are outlined below:

1. After dissection, transfer the samples into a sterile 1.5 mL microcentrifuge tube and fix them in a 4 % PFA solution for 30 min at room temperature.
  **Tip:** To prevent samples from sticking to the wall of a dropper during the transfer process, rinse the dropper with heptane before moving samples from the dissection dish to a microcentrifuge tube.
2. Wash and permeabilize the samples three times with PBSTx (Table 3) for 10 mins each.
3. Enhance sample permeability by employing 1 µg/mL PK for an optimal duration. If the optimal duration is not yet established, it is advised to test several time intervals to determine the best condition. In this protocol, we demonstrated to identify the optimal condition for embryos at different stages. The tested conditions are listed below:

(a) We set up four treatment conditions to validate the optimal time duration for PK treatment in embryos, starting from the 10-minute interval, the reported minimum duration for enhancing tissue permeability [11], and progressed to 20, 40 and 60 min.
(b) For the adult tissue, the salivary glands in this study, we set up five treatment conditions. In particular, we also include a group with no PK treatment as the tissue permeability has not been reported before.
  **Critical:** PK from different sources or batches may exhibit varying enzyme activity. Once an optimal duration is determined, it is suggested to consistently use the same batch of PK. However, if a different batch or source of PK is selected to be used, it is recommended to re-evaluate the optimal duration.

4. Wash the samples three times with 2 mg/mL glycine, for 5 mins each, to inhibit PK activity.
5. Wash the samples twice with PBSTx, for 10 mins each, to remove residual glycine.
6. Re-fix samples with 4 % PFA for 15 mins to maintain the overall integrity of samples, as they could become fragile after PK treatment.
7. Dehydrate the samples gradually with a methanol: PBSTx buffer at three ratios: 1:3, 1:1, and 3:1, for 10 mins each in sequence.
  **Critical:** If a methanol-sensitive enzyme is planned to use as the secondary antibody or if the phalloidin staining is required, please omit the dehydration step.
8. Dehydrate the samples completely with methanol overnight at −20 °C.
  **Pause point:** After the full dehydration of samples, they can be preserved in methanol at −20 °C up to one month.
9. Rehydrate the samples gradually with a PBSTx: methanol buffer at three ratios: 1:3, 1:1, and 3:1, for 10 mins each in sequence.
10. Wash the samples with PBSTx for 10 mins to remove residual methanol.

#### Signal detection and enhancement

For pea aphids, the use of blocking solution on rehydrated embryos and salivary glands is necessary to obtain optimal results with low noise. This critical step minimizes non-specific or background staining, and takes place before sample incubation with primary and secondary antibodies. In a previous study, we demonstrated that the commercialized blocking reagents in the DIG Wash and Block Buffer Set (Sigma-Aldrich) (Table 4) can significantly reduce background signals compared to the normal goat serum (NGS)/bovine serum albumin (BSA) approach in pea aphids. Nevertheless, we recommend comparing signal specificity between commercialized blocking reagents and the traditional NGS/BSA method when performing antibody staining in other aphid species. For details on signal detection and enhancement steps, please refer to the protocols outlined by Lin and Chang (2016) [11].

## Method validation

### Tissue dissection

The experimental steps outlined above serve as a guide for performing an immunostaining experiment on asexual pea aphids, covering the process from tissue dissection to signal development. We demonstrate how to dissect ovaries and salivary glands while preserving the integrity of their structures. The common steps for dissecting both organs include removing outer appendages, opening the body, and separating target tissues from non-target tissues. These principles can be applied to the dissection of other tissues as well. The differences in dissecting distinct tissue types will depend on their specific locations and the methods used to remove associated non-target tissues.

### Proteinase K treatment

Using proteinase K (PK) to digest tissue has become a common method to increase tissue permeability when performing both whole-mount *in situ* hybridization (WISH) and immunostaining in animals. However, the necessity of PK treatment in these technologies is optional. For instance, PK digestion has been reported in WISH application on insect embryos of *Apis mellifera* (honey bee) [25], *Drosophila melanogaster* (fruit fly) [26], *Gryllus bimaculatus* (cricket) [27], *Schistocerca gregaria* (grasshopper) [28], and *Tribolium castaneum* (beetle) [29]. In contrast, the requirement of PK is not mentioned in immunostaining protocols for these insect species [[30], [31], [32]]. Conversely, in *Acyrthosiphon pisum* (pea aphid), we find that PK treatment is not essential for the WISH protocol but it is required for immunostaining on middle and late-stage embryos [11]. Taken together, it indicates that the necessity of PK treatment for probe or antibody penetration depends on composition and thickness of tissues.

**Fig. 4:**
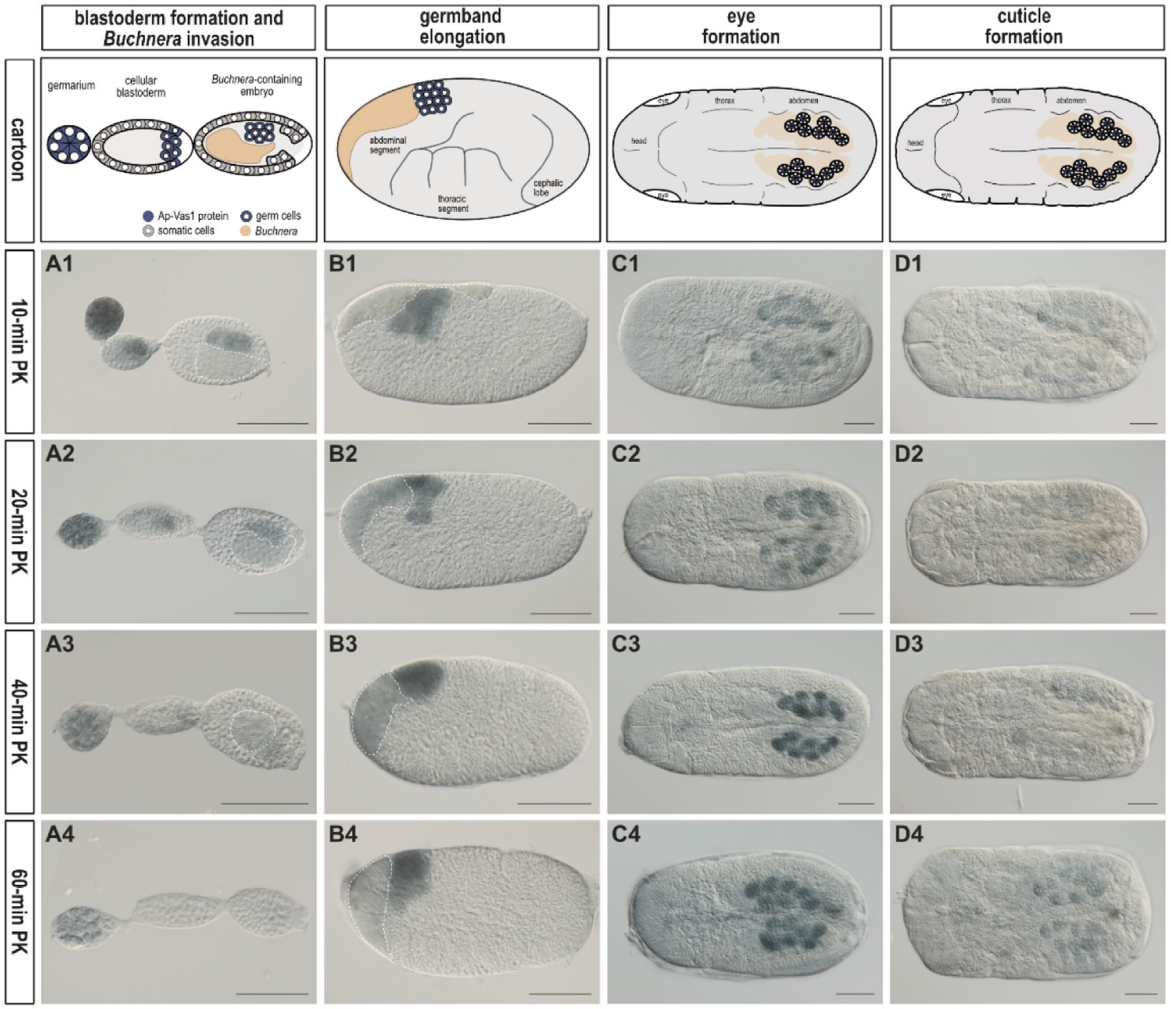
Optimization of proteinase K treatment for enhancing Ap-Vas1 signals in embryos at various stages. Blastoderm formation and germband elongation occur before katatrepsis, while the formation of eyes and cuticle occurs after katatrepsis. In embryos of asexual pea aphids, the anterior orientation of embryos is to the right before katatrepsis and to the left after katatrepsis. The cartoon illustrations depict the developmental features of embryos, with color keys displayed above panel A1. Embryos were subjected to proteinase K treatment at concentrations of 1 µg/mL for 10, 20, 40, and 60 min, respectively. The germline-specific protein Ap-Vas1 was visualized as blue-black signals. (A1-A4) Embryos undergoing blastoderm formation and invasion of *Buchnera* endosymbionts are presented. The signal intensity of Ap-Vas1 significantly decreases in embryos incubated with proteinase K for >40 min (see A3 and A4). (B1-B4) Embryos undergoing germband elongation are presented in lateral view. Background staining is detectable in the *Buchnera* endosymbionts (encircled by a white dotted line in panels A1-A4 and B1-B4). (C1-C4) Embryos with morphologically identifiable eyes are presented in dorsal view. In panels B1-B4 and C1-C4, the intensity of Ap-Vas1 signals is elevated in embryonic germ cells incubated with proteinase K for >40 min (see B3, B4; C3, C4). (D1-D4) Embryos covered with cuticle are presented in dorsal view. Ap-Vas1 signals were barely detected in embryos treated with proteinase K for varying incubation lengths. Abbreviations: min, minute; PK, proteinase K. All images have scale bars of 50 µm.
(2) In embryos with multiple cellular layers, such as those undergoing germband elongation (Fig. 4 B1–B4), both 40-minute and 60-minute PK incubation were shown to be optimal (Fig. 4B3 and B4). However, for embryos with morphologically identifiable eyes (Fig. 4 C1–C4), a 40-minute PK incubation (Fig. 4C3) was more effective than a 60-minute incubation (Fig. 4C4) due to the stronger staining background with the 60-minute incubation, which could interfere with the identification of target signals in the germ cells.
(3) For embryos fully covered with cuticle, prolonged treatment with PK up to 60 min did not significantly enhance the staining signals (Fig. 4D4). This hindrance of antibody penetration could not be resolved by extending the treatment up to 3 h or raising the PK concentration to 50 µg/mL for 30-minute incubation. It is likely that PK has digested the cuticle protein, but the polysaccharide chitin—a non-protein component of the cuticle [33]—remains intact, resisting the penetration of the antibody.
(4) The dissected salivary glands were incubated with PK for 60 min to exhibit the most intensive and specific signals of C002 (Fig. 5E), a known salivary gland protein in pea aphids [34], compared to those incubated for 10, 20, and 40 min (Fig. 5B–D). On the other hand, salivary glands devoid of PK treatment only display background signals (Fig. 5A).

In addition to the step-by-step protocols provided above, we specifically explain the rationale for establishing the optimal conditions:

(1) When ovarioles were treated with PK (1 µg/mL) for 10 min, as previously reported [11], the intensity of Ap-Vas1 signals in germaria, oocytes, and embryos undergoing *Buchnera* invasion was stronger (Fig. 4A1) compared to those incubated with PK for 20, 40, and 60 min (Fig. 4 A2–A4). This suggests that prolonged PK incubation leads to the digestion of endogenous proteins like Ap-Vas1 (Fig. 4 A2–A4) and damages the morphological integrity of thin tissue layers, such as germaria and cellular blastoderm (Fig. 4A4).

**Fig. 5:**
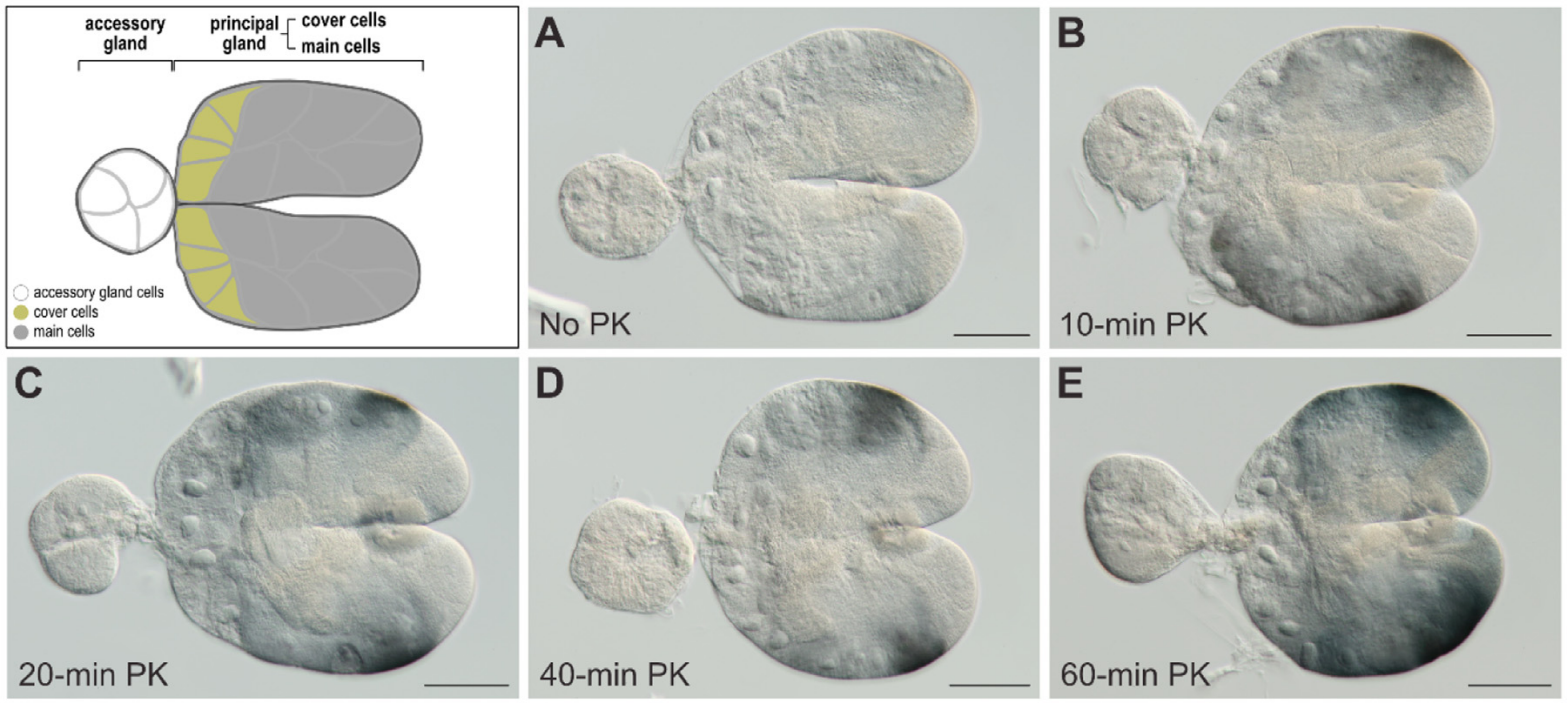
Optimization of proteinase K treatment for detecting C002 protein in the salivary glands. The salivary glands in all panels are positioned with the anterior to the left. The glands were subjected to proteinase K treatment at concentrations of 1 µg/mL for 0, 10, 20, 40, and 60 min, respectively, and C002 protein expression was visualized as blue-black signals. A schematic representation with color keys illustrates the structure of the salivary glands, comprising a principal gland and an accessory gland. Cover cells are situated in the anterior region of the principal gland but main cells occupy the remainder of the gland. (A) In the absence of proteinase K treatment, signals were scarcely detected in both the principal and accessory glands. (B-E) Upon treatment, signals were more prominently detected in the principal gland. Abbreviations: min, minute; PK, proteinase K. All images have scale bars of 50 µm.

## Conclusion

In summary, optimizing PK treatment is crucial for enhancing tissue permeability in asexual pea aphids. The optimal conditions for PK treatment can vary depending on the batch of PK and antibodies used. Furthermore, PK digestion may need to be tailored for different reproductive morphs of each aphid species, even when targeting the same tissues and organs. Our experiments demonstrate that both PK concentration and digestion duration are key factors that require adjustment to ensure effective antibody penetration into intact tissues through precise dissection.

## CRediT authorship contribution statement

**Chun-wei Lai:** Methodology, Validation, Investigation, Writing – original draft, Writing – review & editing, Visualization. **Gee-Way Lin:** Methodology, Resources, Writing – review & editing. **Wen-Chih Lee:** Conceptualization, Validation, Writing – review & editing, Supervision. **Chun-che Chang:** Conceptualization, Writing – original draft, Writing – review & editing, Visualization, Supervision, Project administration, Funding acquisition.

## Declaration of competing interest

The authors declare that they have no known competing financial interests or personal relationships that could have appeared to influence the work reported in this paper.

## Data Availability

No data was used for the research described in the article.
